# Speciation and evolution of growth form in *Adesmia* D. C. (Dalbergieae, Fabaceae): the relevance of Andean uplift and aridification

**DOI:** 10.3389/fpls.2024.1403273

**Published:** 2024-10-15

**Authors:** Fernanda Pérez, Nicolás Lavandero, Luis Felipe Hinojosa, Mauricio Cisternas, Daniela Araneda, Nicolás Pinilla, Valeska Moraga

**Affiliations:** ^1^ Facultad de Ciencias Biológicas, P. Universidad Católica de Chile, Santiago, Chile; ^2^ Departamento de Ecología, Facultad de Ciencias, Universidad de Chile, Santiago, Chile; ^3^ Instituto de Investigaciones Agropecuarias (INIA), La Cruz, Chile

**Keywords:** Andes, Atacama Desert, biogeography, climatic niche, diversification, life history strategy, South American Arid Diagonal

## Abstract

The Andean uplift and the concomitant aridification drove the rapid diversification of several plant lineages that were able to colonize warmer and drier habitats at low elevations and wetter and colder habitats at high elevations. These transitions may be facilitated by shifts in plant strategies to cope with drought and cold, which in turn can trigger episodes of accelerated species diversification. Here, we used four nuclear DNA markers to infer phylogenetic relationships of 80 *Adesmia* species of annuals, perennial herbs, shrubs and small shrubs that occur in Chile and Argentina. We reconstructed ancestral states for area, climatic niche and growth form to explore how Andean uplift and aridification promoted *Adesmia* diversification. We also performed logistic and linear regression analyses between different components of growth form (life span, woodiness and plant height) and climate. Finally, we estimated speciation rates across the phylogeny. Our results suggest that the ancestor of Chilean *Adesmia* was a perennial herb that probably originated in the high Andes of northern and central Chile. The low elevations of Central Chile were colonized in the late Miocene, whereas the high latitudes of Patagonia and the hyperarid coastal Atacama Desert were colonized repeatedly since Pliocene by lineages with different growth forms. Multiple and bidirectional transitions between annual and perennial habits and between herbaceous and woody habits were detected. These shifts were not correlated with climate, suggesting that the different growth forms are alternative and successful strategies to survive unfavorable seasons of both desert and high Andes. Net diversification analysis indicated a constant rate of diversification, suggesting that the high species diversity of *Adesmia* that occur in Chile is due to a uniform speciation process rather than to accelerated episodes of speciation.

## Introduction

The central and southern Andes have a high diversity of plant species (Pérez-Escobar et al., 2022). The history of this diverse flora is linked to the orogeny of the Andes and the development of the South American Arid Diagonal (Simpson, 1983; Antonelli et al., 2009; Luebert and Weigend, 2014), a strip of arid climate that extends along the Andes from Peru to Argentinian Patagonia (Villagrán and Hinojosa, 1997; Abraham et al., 2020). Aridification occurred gradually during the Oligocene and Miocene as a consequence of the strong rain shadow produced by the uplift of the Andes (Dunai et al., 2005). The establishment of a phase of global climate cooling since the middle Miocene (Zachos et al., 2001) and the reinforcement of the cold Humboldt Current in the Pliocene intensified aridification (Hinojosa and Villagrán, 1997; Hartley et al., 2005; Garreaud et al., 2010).

The South American Arid Diagonal forms a biogeographic corridor that has allowed the expansion and diversification of plant genera of different biogeographic origins (Simpson, 1983; Antonelli et al., 2009; Luebert and Weigend, 2014; Glade-Vargas et al., 2021). In the high Andes, plant lineages that have migrated from high latitudes tracking cold-humid climates coexist with lineages that have shifted repeatedly between warmer and drier habitats at low elevations and colder and wetter habitats at high elevations (Antonelli, 2015). Transitions between habitats may be accompanied by shifts in the strategy of plants that allow surviving periods of extreme cold or drought (Ogburn and Edwards, 2015; Pérez et al., 2020); or alternatively, might be favored by the preexistence of “enabler” traits that allow species to cope with both conditions (Harvey and Pagel, 1991; Edwards and Donoghue, 2013; Zanne et al., 2014).

Plants have evolved different strategies that permitted them to survive unfavorable seasons (Grime, 1977; Stearns, 2000). The annual life strategy is part of a fast development strategy that allows plants to complete the life cycle within the favorable season of a single year (Evans et al., 2005; Kooyers, 2015; Friedman and Rubin, 2015; Pérez et al., 2020). Annuals are favored in hot and dry seasonal climates (Friedman, 2020; Boyko et al., 2023), but tend to be excluded from high elevation habitats, where short growing seasons and repeated frosts inhibit seedling establishment and reduce the probability of completing a life cycle in one season (Bliss, 1971; Korner, 2003; Givnish, 2015). Perennial plants also have adaptations that allow survival of unfavorable seasons. Perennial herbs can shed aboveground tissues during unfavorable seasons, leaving belowground buds in rhizomes (woody underground stems) or specialized reserve organs (such as bulbs, tubers or corms) that enable resprouting (Raunkiaer, 1934; Bliss, 1971) and allow plants to survive in cold and dry conditions (Howard et al., 2019). Woody plants can shed leaves and protect buds with scale-like modified leaves or become smaller, leaving buds close to the ground (Bliss, 1971; Korner, 2003; Zanne et al., 2014). According to these strategies, plants can be classified in those that persist as seeds or underground storage organs and those that have perennial buds at, near or above the soil surface (Raunkiaer, 1934; Taylor et al., 2023).

Accelerated rates of diversification can be triggered by shifts in traits related with plant life history strategies, including life span (Givnish, 2010; Friedman, 2020), woodiness (Nürk et al., 2019), plant size (Boucher et al., 2017) and reproductive strategy (de Vos et al., 2014). Annual and herbaceous clades often have more species than their sister woody clades (Givnish, 2010), indicating that the shorter generation time of herbs can accelerate speciation (Smith and Donoghue, 2008). Likewise, small plants have higher diversification rates than do larger plants (Boucher et al., 2017). Shifts from annual to perennial life history strategy have been proposed as key innovations that trigger accelerated species diversification in high elevation habitats (Drummond et al., 2012; Hughes and Atchison, 2015; Nevado et al., 2016; but see Givnish, 2015). Evolution of secondary woodiness from herbaceous ancestors also appears to act as a key innovation driving plant radiations on island systems (Nürk et al., 2019).

**Figure 1:**
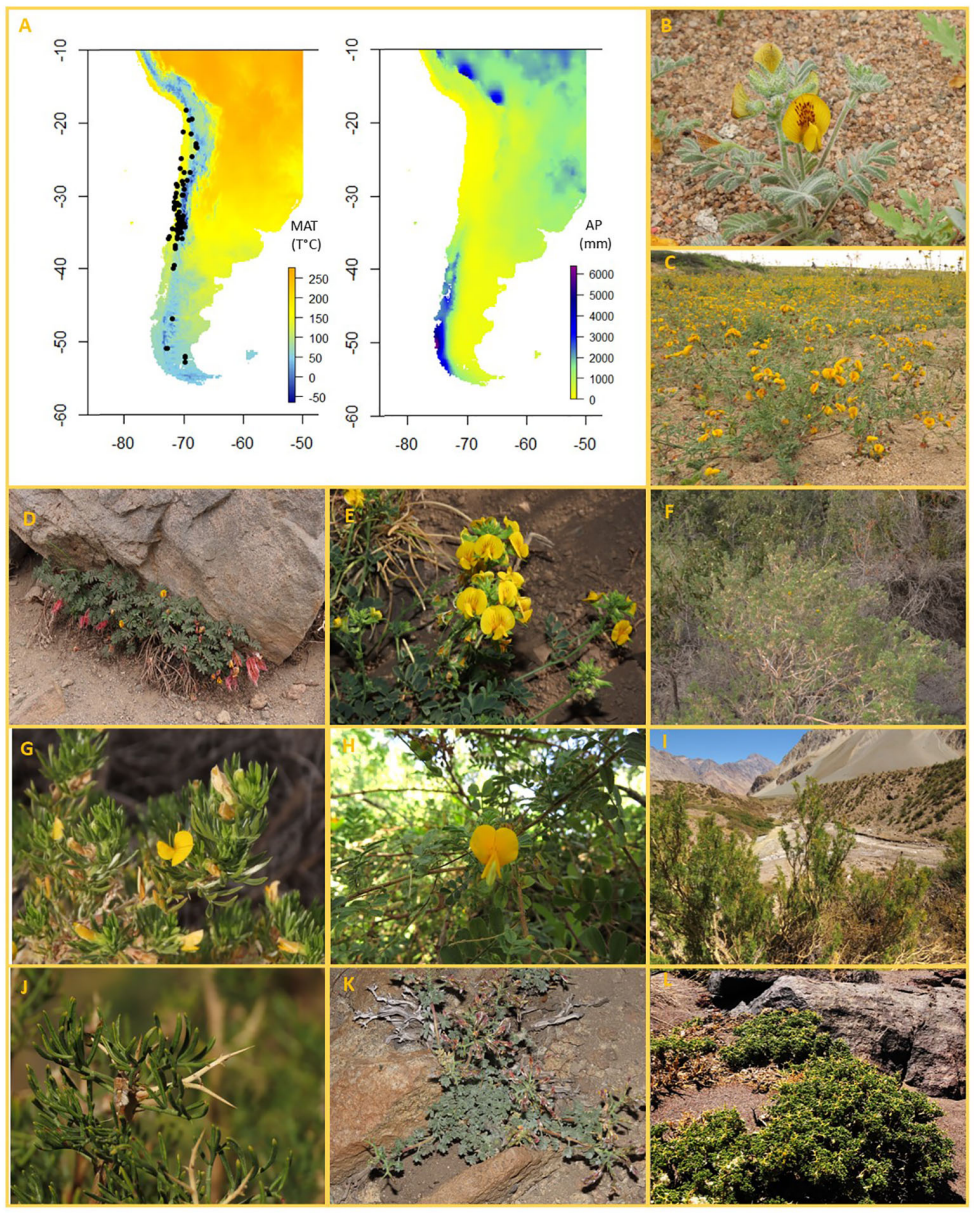
**(A)** Distribution of studied species of *Adesmia* in relation to mean annual precipitation (left) and mean annual temperature (right). **(B-L)** Images of 9 species of *Adesmia* showing the wide variety of growth forms found in the genus. **(B, C)** Annuals: **(B)** *A. eremophila*, **(C)** *A tenella*. **(D, E)** Perennial herbs: **(D)** *A longipes*, **(E)** *A. coronilloides*. **(F-J)** Shrubs (larger than 25 cm): **(F, G)** *A. loudonia* (unarmed shrub); **(H)**. *A. elegans* (unarmed shrub); **(I, J)** *A. pinifolia* (spiny shrub). **(K, L)** Dwarf shrubs: **(K)** *A. hirsuta*; **(L)**. *A. hemisphaerica*.

The Andean uplift and aridification drove the rapid diversification of several plant lineages, including some of the most species-rich genera of Fabaceae (Kenicer et al., 2005; Hughes and Eastwood, 2006; Scherson et al., 2008). In southern South America, the richest genus of Fabaceae is *Adesmia* DC. (Dalbergieae, Faboideae), which comprises around 230 species of annuals, perennial herbs, small cushions and shrubs (**Figure 1**) grouped in 43 series and two subgenera: *Acanthadesmia* (that includes all spiny shrubs) and *Adesmia* (that includes all unarmed forms) (Burkart, 1967). Species grow mainly along the Arid Diagonal from northern Peru to Patagonia, with some species extending to Southern Brazil. *Adesmia* has two centers of diversification, one in Chile and the other in Argentina (Burkart, 1967). Both areas have been proposed as centers of origin, with the Andes acting as a vicariant barrier (Mihoc, 2012) or as a biogeographic corridor from south to north (Burkart, 1967). A phylogenetic study has been conducted for *Adesmia* sect. *psoraleoides*, a monophyletic series endemic to southern Brazil (Iganci et al., 2013). This study, which included some species from other regions, found a north to south pattern of diversification, suggesting an expansion from arid regions of Bolivia and Chile to Patagonia, followed by colonization of southern Brazil.

**Figure 2:**
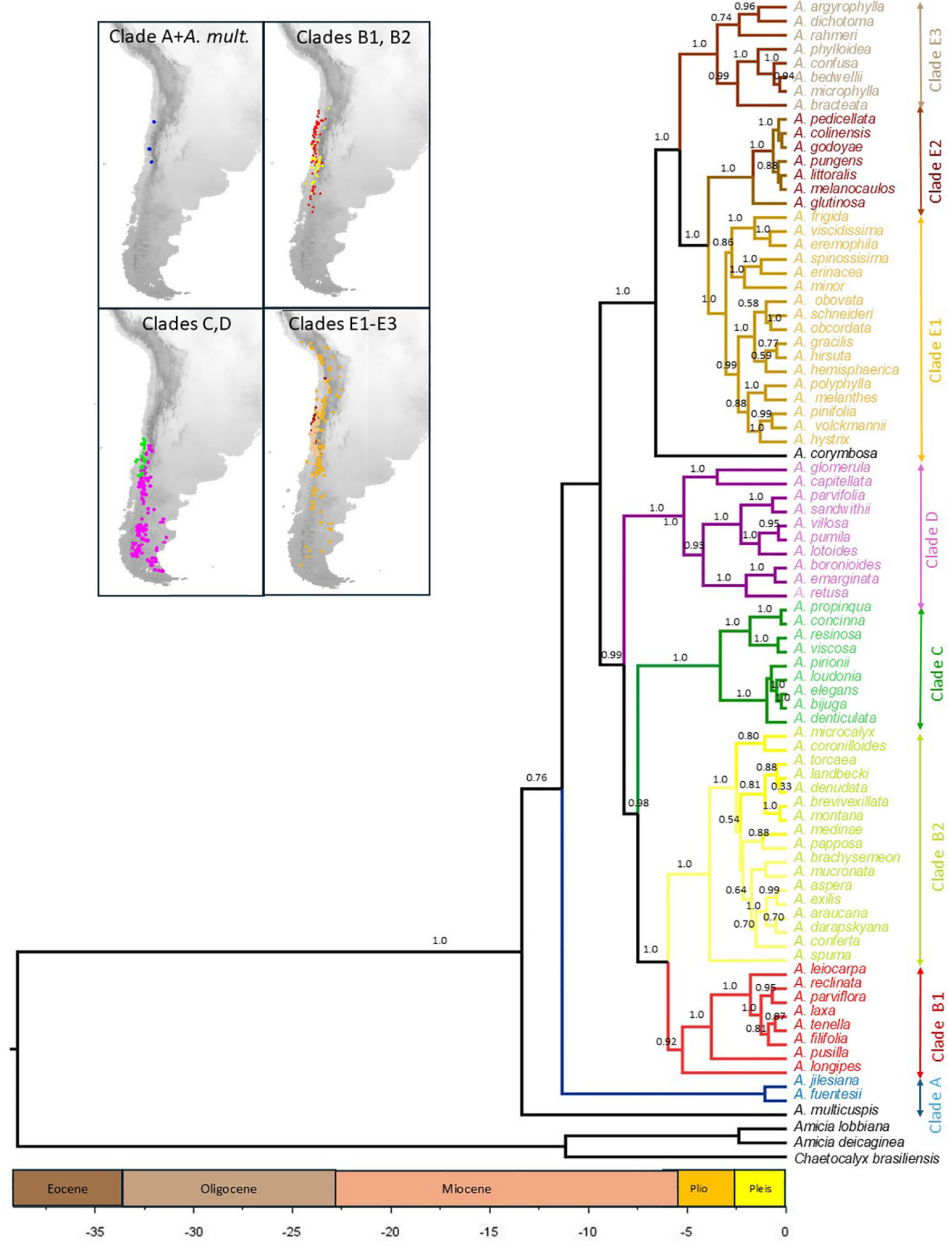
Time-calibrated phylogeny of 80 species of *Adesmia* that grow in Chile based on four nuclear DNA sequences obtained in BEAST. Three species of the related genera *Amicia* and *Chaetocalyx* were included as outgroups. Divergence time is given in million years before present. The posterior probabilities of nodes are shown above branches (only values higher than 0.5). Distribution of species of the main lineages (denoted with different colors) are shown in maps.

In this study, we established phylogenetic relationships using four nuclear DNA markers of 80 *Adesmia* species that occur in Chile and Argentina from latitude 18° to 52°S and from sea level to 4500 m elevation (**Figure 2**). We reconstructed ancestral states for area, climatic niche and growth form, to understand how the Andean uplift and the onset of arid and semiarid conditions promoted diversification of *Adesmia*. We also included the presence/absence of spines to explore whether the two subgenera (*Acanthadesmia* and *Adesmia*) are monophyletic. In addition, we examined whether shifts in traits related to plant life history strategy (life span, woodiness and plant height) evolved in relation to climate. Finally, we determined whether the high species diversity of Chilean *Adesmia* is due to uniform speciation, or to events of accelerated speciation triggered by shifts in growth form.

## Materials and methods

### Taxon sampling

We obtained DNA from 80 of the 132 species described for Chile (Rodríguez et al., 2018; **Table 1**). DNA was extracted from leaf material of individuals collected in the field and from herbarium specimens stored at CONC (Herbarium of the Department of Botany, University of Concepción) and SGO (Herbarium of the National Museum of Natural History). As outgroup species, we included *Chaetocalyx brasiliensis* (Vogel) Benth., *Amicia medicaginea* Griseb., and *Amicia lobbiana* Benth.

**Table 1:** List of *Adesmia* species considered in this study.

Voucher	Herb	Species	Author	Subgenus	Series	Distribution
Humaña & al. 20011	CONC	*araucana*	Phil.	*Adesmia*	Araucanae	CHI
NL-1803	SGO	*argyrophylla*	Phil.	*Acanthadesmia*	Argyrophyllae	CHI
NL-1463	SGO	*aspera*	Gillies ex Hook. & Arn.	*Adesmia*	Papposae	ARG, CHI
NL-1600	SGO	*bedwellii*	Skottsb.	*Acanthadesmia*	Glutinosae	CHI
Espejo & San Martin 10	CONC	*bijuga*	Phil.	*Adesmia*	Loudoniae	CHI
Maria Fernanda Pérez *s.n.*	SGO	*boronioides*	Hook.f.	*Adesmia*	Balsamicae	ARG, CHI
Mauricio Cisternas s.n.	SGO	*brachysemeon*	Phil.	*Adesmia*	Papposae	CHI
NL-1788	SGO	*bracteata*	Hook. & Arn.	*Adesmia*	Bracteatae	CHI
Arroyo & al. 994690	SGO	*brevivexillata*	Burkart	*Adesmia*	Araucanae	CHI
NL-1561	SGO	*capitellata*	(Clos) Hauman	*Adesmia*	Capitellatae	ARG, CHI
Macaya & al. 359	CONC	*colinensis*	(Phil. ex Reiche) Martic.	*Adesmia*	Argenteae	CHI
Macaya & Teillier *s.n.*	CONC	*concinna*	Phil.	*Adesmia*	Balsamicae	CHI
Garcia & al. 2758	SGO	*conferta*	Hook. & Arn.	*Adesmia*	Confertae	CHI
Landrum 11504	SGO	*confusa*	Ulibarri	*Acanthadesmia*	Microphyllae	CHI
NL-1495	SGO	*coronilloides*	Gillies ex Hook. & Arn.	*Adesmia*	Coronilloides	ARG, CHI
NL-1187	SGO	*corymbosa* var. *corymbosa*	Clos	*Adesmia*	Longisetae	ARG, CHI
Garcia & al. 3489	SGO	*darapskyana*	(Phil. ex Reiche) Martic.	*Adesmia*	Papposae	CHI
NL-0154	SGO	*denticulata*	Clos	*Adesmia*	Denticulatae	CHI
Garcia & al. 628	SGO	*denudata*	Phil.	*Adesmia*	Papposae	CHI
NL-1448	SGO	*dichotoma*	Clos	*Adesmia*	Argenteae	CHI
NL-0157	SGO	*elegans*	Clos	*Adesmia*	Denticulatae	CHI
NL-1489	SGO	*emarginata*	Clos	*Adesmia*	Balsamicae	ARG, CHI
NL-1449	SGO	*eremophila*	Phil.	*Adesmia*	Longisetae	CHI
Diaz & Latorre 625	CONC	*erinacea*	Phil.	*Acanthadesmia*	Microphyllae	ARG, CHI
NL-1462	SGO	*exilis*	Clos	*Adesmia*	Longisetae	ARG, CHI
NL-1754	SGO	*filifolia*	Clos	*Adesmia*	Leiocarpae	CHI
Teillier & Medrano 8354	CONC	*frigida*	Phil.	*Acanthadesmia*	Frigidae	CHI
Mauricio Rebolledo *s.n.*	SGO	*fuentesii*	G.F. Grandjot	*Adesmia*	Fuentesii	CHI
NL-1454	SGO	*glomerula*	Clos	*Adesmia*	Glomerulae	ARG, CHI
NL-1321	SGO	*glutinosa*	Hook. & Arn.	*Acanthadesmia*	Glutinosae	CHI
Claire de Schrevel 395	SGO	*godoyae*	(Phil. ex Reiche) Martic.	*Acanthadesmia*	Glutinosae	CHI
NL-1472	SGO	*gracilis*	Meyen ex Vogel	*Acanthadesmia*	Microphyllae	ARG, CHI
NL-1563	SGO	*hemisphaerica*	Hauman	*Acanthadesmia*	Subterraneae	ARG, CHI
NL-1584	SGO	*hirsuta*	Phil.	*Acanthadesmia*	Microphyllae	CHI
NL-1825	SGO	*hystrix*	Phil.	*Acanthadesmia*	Microphyllae	CHI
NL-1527	SGO	*jilesiana*	Burkart	*Adesmia*	Fuentesii	ARG, CHI
García 3723	CONC	*landbeckii*	Phil.	*Adesmia*	Longisetae	CHI
NL-1755	SGO	*laxa*	Clos	*Adesmia*	Confertae	CHI
NL-0783	SGO	*leiocarpa*	Hook. & Arn.	*Adesmia*	Leiocarpae	CHI
NL-1424	SGO	*littoralis*	Burkart	*Acanthadesmia*	Argyrophyllae	CHI
NL-1466	SGO	*longipes*	Phil.	*Adesmia*	Longipedes	ARG, CHI
NL-1198	SGO	*lotoides*	Hook.f.	*Adesmia*	Lotoides	ARG, CHI
NL-0555	SGO	*loudonia*	Hook. & Arn.	*Adesmia*	Loudoniae	CHI
Saldivia & Larrain 1384	CONC	*medinae*	(Phil. ex Reiche) Ulibarri	*Adesmia*	Longisetae	CHI
NL-1410	SGO	*melanocaulos*	Phil.	*Acanthadesmia*	Microphyllae	CHI
Teillier & Mella 6423	CONC	*melanthes*	Phil.	*Acanthadesmia*	Microphyllae	CHI, PER
Garcia & al. 2346	SGO	*microcalyx*	Phil.	*Adesmia*	Papposae	CHI
NL-1423	SGO	*microphylla*	Hook. & Arn.	*Acanthadesmia*	Microphyllae	CHI
Prado 3	CONC	*minor*	(Hook. & Arn.) Burkart	*Acanthadesmia*	Subterraneae	ARG, CHI
Pozner & Medina 624	SGO	*montana*	Phil.	*Adesmia*	Longisetae	CHI
Garcia & al. 3167	SGO	*mucronata*	Hook. & Arn.	*Adesmia*	Papposae	CHI
Claire de Schrevel 338	SGO	*multicuspis*	Clos	*Adesmia*	Tenellae	CHI
Luebert & Teillier 2335	CONC	*obcordata*	Clos	*Acanthadesmia*	Microphyllae	ARG, CHI
	SGO	*obovata*	Clos	*Acanthadesmia*	Microphyllae	ARG, CHI
Madrid & Larrain 148	CONC	*papposa* var. *papposa*	(Lag.) DC.	*Adesmia*	Papposae	ARG, CHI
NL-1710	SGO	*parviflora*	Clos	*Adesmia*	Leiocarpae	CHI
G. Rojas & Saldivia *s.n.*	SGO	*parvifolia*	Phil.	*Adesmia*	Lanatae	ARG, CHI
NL-1653	SGO	*pedicellata*	Hook. & Arn.	*Acanthadesmia*	Glutinosae	CHI
Teillier 5921	CONC	*phylloidea*	Clos	*Adesmia*	Phylloideae	CHI
NL-1560	SGO	*pinifolia*	Gillies ex Hook. & Arn.	*Acanthadesmia*	Microphyllae	ARG, CHI
Abello & Videla *s.n.*	SGO	*pirionii*	I.M. Johnst.	*Adesmia*	Loudoniae	CHI
Muñoz & Moreira 4978	SGO	*polyphylla*	Phil.	*Acanthadesmia*	Microphyllae	BOL, CHILE
Moreira 914	SGO	*propinqua*	Clos	*Adesmia*	Balsamicae	CHI
E. Dominguez 32	CONC	*pumila*	Hook.f.	*Adesmia*	Pumilae	ARG, CHI
NL-1662	SGO	*pungens*	Clos	*Acanthadesmia*	Microphyllae	CHI
Schneider & Huertas 2848	CONC	*pusilla*	Phil.	*Adesmia*	Leiocarpae	CHI
NL-0648	SGO	*rahmeri*	Phil.	*Adesmia*	Hispidulae	ARG, CHI
NL-1421	SGO	*reclinata*	Muñoz	*Adesmia*	Confertae	CHI
NL-0504	SGO	*resinosa*	(Phil. ex Reiche) Martic.	*Adesmia*	Balsamicae	CHI
Teillier & al. 7390	CONC	*retusa*	Griseb.	*Adesmia*	Retusae	ARG, CHI
Elvebakk 96:658	CONC	*sandwithii*	Burkart	*Adesmia*	Lanatae	ARG, CHI
NL-1564	SGO	*schneideri*	Phil.	*Acanthadesmia*	Microphyllae	ARG, CHI
Munoz & Moreira 4980	SGO	*spinosissima*	Meyen	*Acanthadesmia*	Microphyllae	ARG, BOL, CHI, PER
Claire de Schrevel 08	SGO	*spuma*	Werderm. ex Burkart	*Adesmia*	Spumae	ARG, CHI
NL-1778	SGO	*tenella* var. *macrocarpa*	Hook. & Arn.	*Adesmia*	Tenellae	CHI
NL-1750	SGO	*torcaea*	Phil.	*Adesmia*	Longisetae	CHI
E. Dominguez 107	CONC	*villosa*	Hook.f.	*Adesmia*	Pumilae	ARG, CHI
Macarena M.Villalobos *s.n.*	SGO	*viscidissima*	I.M. Johnst.	*Adesmia*	Longisetae	CHI
Eyzaguirre 29	SGO	*viscosa*	Gillies ex Hook. & Arn.	*Adesmia*	Balsamicae	CHI
Baeza et al. 3411	CONC	*volckmannii*	Phil.	*Acanthadesmia*	Microphyllae	ARG, CHI

The voucher, herbarium, subgenera and series of each species according to Burkart (1967) and the distribution are shown. GenBank accessions numbers are in **Supplementary Data Sheet**. ARG, Argentina; BOL, Bolivia; CHI, Chile; PER, Perú.

### DNA extraction, amplification, and sequencing

Genomic DNA was extracted using the DNeasy Plant Kit (Qiagen, Valencia, CA, United States). PCR amplification was performed for the internal transcribed spacer region (ITS), the external transcribed spacer region (ETS), and the auxin-independent growth (AIGP) gene using previously published primers (Ariati et al., 2006; Choi et al., 2004; **Table 2**). Additionally, we searched for nuclear single-copy gene using the transcriptome sequences of two species of *Adesmia* obtained by Eshel et al. (2021). We designed primers for ten candidate regions and then we conducted PCR amplification in ten species to test for amplification consistency and specificity. We selected the two most polymorphic regions, one corresponding to U5 small nuclear ribonucleoprotein component (CLO) and the other to the “vacuolar-sorting receptor 1” gene (exons 2 to 7). Sanger sequencing was performed in the Plataforma de Secuenciación y Tecnologías Omicas, Pontificia Universidad Católica de Chile, using the ABI PRISM 3500 xl Genetic Analyzer (Applied Biosystems). All new sequences were deposited in GenBank (**Supplementary Table 1**). Sequences were aligned using the ClustalW algorithm in BioEdit 7.0 (Hall, 1999) and concatenated into a single dataset consisting of a total of 2949 positions. Alignments were manually inspected. Previously, we performed a homogeneity test in PAUP 4.0 (Swofford, 2003) to assess whether multi-copy ribosomal markers (ITS, ETS) and single-copy nuclear genes are congruent. We found that the two datasets are congruent (p=0.17). Given that this test can yield false negatives (Pirie, 2015), we also analyzed the ribosomal and single-copy nuclear genes separately. Then, the resulting phylogenetic trees were examined manually.

**Table 2:** List of primers used in this study.

Region	Forward	Reverse	Reference
ITS	ITS-26SE TAG AAT CCC CGG TTC GCT CGC CGT TAC	ITS-S3 AAC CTG CGG AAG GAT CAT TG	Ariati et al., 2006
ETS	ETS-AcR2 GGG CGT GTG AGT GGT GTT TGG	ETS-18S-IGS CAC ATG CAT GGC TTA ATC TTT G	Ariati et al., 2006
AIGP	AIGP_F CTGATAGGGCCAGGAGGCAGGGAAGA	AIGP_R GTTTTTTAGCATTTGGACGAATGGTTGGT	Choi et al., 2004
2280	ad_2280F CATCACACAAAGGTCCCTCT	ad_2280R CCTCTAGCCGTTACCAAAGT	Own design
3813	ad_3813F ATACTCGCCCACGGAATGTATC	ad_3813R TGAAACCAAAAAGCCTTGGGTG	Own design

### Phylogenetic analyses

Phylogeny was reconstructed using Bayesian Inference (BI) as implemented in MrBayes 3.1.2 (Ronquist et al., 2012) on the combined dataset. Analyses were conducted using four partitions corresponding to each DNA region and a GTR + γ model of evolution, with two independent runs for 10 million generations, sampling every 5,000 generations. We discarded the first 25% of generations as burn-in to construct a 50% majority-rule consensus tree and to obtain posterior probabilities of each node (PP). To estimate species divergence times, we conducted a Bayesian relaxed-clock analysis in BEAST (BE) program (version 1.4.8; Drummond and Rambaut, 2007). We used the age of the most recent common ancestor of *Adesmia* and *Chaetocalyx* (40.9 ± 4 Mya) as a calibration point according to Simon et al. (2009). MCMC chains were run for 50 million generations, sampling every 5,000 generations. The time-scaled maximum clade credibility (MCC) tree was then identified using TreeAnnotator. We discarded the first 25% of generations as burn-in to obtain posteriori probabilities of each node.

### Climatic niche

We estimated the realized climatic niche of each species using the maximum entropy approach with MAXENT (Phillips et al., 2006), incorporating 19 bioclimatic and topographic variables from the World Climate database (Hijmans et al., 2005) at 30 arc seconds of resolution one km (30s) resolution (~1 Km at the equator). We compiled distribution information for each species from GBIF and newly collected field data. These occurrences were verified with each species distribution reported in Rodriguez et al. (2018). Occurrences outside the known distribution range were eliminated. Background points were randomly selected within the area enclosed by a minimum convex polygon comprising all species records. Occurrence data were partitioned 100 times into training and test datasets (80% and 20%, respectively) for model evaluation using the operating characteristic curve (AUC). We focused on mean annual temperature (MAT) and annual precipitation (AP) because these variables describe better the diversity of habitats occurring along the latitudinal and elevational gradient where *Adesmia* is distributed. Probability distributions derived from MAXENT were used to obtain predicted niche occupancy profiles of each species with respect to mean annual temperature (MAT) and mean annual precipitation (AP). We estimated the weighted mean of each climatic variable (w-MAT, w-AP). All analyses were conducted using the R-package phyloclim (Heibl, 2011). The ancestral states of w-AP and w-MAT were reconstructed using the MCC tree recovered from BEAST analysis and the maximum likelihood method of Schluter et al. (1997) under a Brownian model of evolution implemented in the R package phytools (Revell, 2012).

### Evolutionary shifts in growth forms

Growth forms of *Adesmia* were classified as annuals (persist as seed), perennial herbs (shed aboveground tissues during unfavorable seasons, leaving resistant buds in rhizomes) and shrubs (**Supplementary Table 2**). The ancestral states of growth form were reconstructed using the Markov Chain Monte Carlo (MCMC) approach implemented in BayesTraits v4 (Meade and Pagel, 2023), which takes into account phylogenetic uncertainty (Pagel and Meade, 2006). These analyses were conducted with 1000 trees randomly sampled from the posterior of the BEAST analysis. We performed a likelihood ratio test (LR) to compare the goodness of fit of an equal rates model with an unequal rates model. We selected the first model because it did not lead to a significant reduction in likelihood (Log marginal likelihood: unequal rates model = -43.2; equal rates model = -44.8; LR=3.2; Chi_5_ = 3.6, p=0.39). We used this model to estimate the average probability of each possible growth form (annual herb, perennial herb, or shrub) for each node based on 1,000 sampled trees. Additionally, we analyzed the presence of spines, a key trait that has been used to separate the currently recognized subgenera *Adesmia* and *Acanthadesmia*.

We also examined whether shifts in different components of the growth form (life span, woodiness, and plant height) evolved in relation to climate, using two approaches. First, we reconstructed the ancestral states for w-MAT and w-AP using the lambda model that incorporates a scaling parameter (λ) to account for the effects of phylogenetic relationships on trait evolution. Previously, we compared the lambda model with a Brownian (BM), early burst (EB) an Ornstein-Uhlenbec (OU) model using the Akaike Information Criterion (AIC). We selected the first model for both climatic variables because it had the lowest AIC values (w-MAT: AIC lambda=175, AIC BM=186, AIC OU 186, AIC EB=187; w-AP: AIC lambda=1100; AIC BM=1127, AIC OU= 1129, AIC EB=1129). Second, for discrete traits, life span (annual/perennial) or woodiness (herb/shrub), we fitted a phylogenetic logistic regression model using w-MAT and w-AP as continuous predictor variables in the R package phylolm (Ho and Ane, 2014). y. For continuous traits (plant height), we conducted a phylogenetic linear regression using w-MAT and w-AP as predictor variables.

### Ancestral area reconstruction

We reconstructed the ancestral distributions of *Adesmia* using several evolutionary process models implemented in the BioGeoBEARS R package (Matzke, 2014) and the consensus tree recovered from BEAST analysis. BioGeoBEARS implemented several anagenetic and cladogenetic process models, including the LaGrange Dispersal-Extinction Cladogenesis Model (DEC) (Ree and Smith, 2008), a likelihood version of DIVA (DIVALIKE), and BAYAREA (Yu et al., 2015). A jump parameter (j) can also be added to any of these models to account for speciation processes mediated by founder events (Matzke, 2014). Models were compared using the Akaike Information Criterion (AIC). We considered five areas: (D) Desert, corresponding to low to mid elevations of northern Chile (18 to 30°S); (N) Northern Chilean High Andes (up to 2000 m, 18 to 30°S); (M) Mediterranean region, corresponding to low to mid elevations of central and southern Chile (30 to 40°S); (C) Central Chilean High Andes: high elevations of central Chile (30 to 40°S) and (P) Patagonia, cold steppes at high-latitudes in Patagonia (southern 40°S). A maximum of two areas was allowed for reconstructions.

### Diversification analyses

To estimate whether the high species diversity of *Adesmia* is due to uniform divergence or events of accelerated speciation, we estimated speciation rates across the *Adesmia* phylogeny using a time-dependent model implemented in BAMM v.2.5 with expected number of shifts set to one and sampling probability set to 0.64 (corresponding to the proportion of Chilean species sampled). Four Markov Chains Monte Carlo (MCMC) runs were performed with 50 million generations, sampling parameters every 5,000 generations. Diversification rates and rate shift configurations were plotted using the BAMMtools R-package (Rabosky et al., 2014).

## Results

### Molecular phylogeny

**Figure 3:**
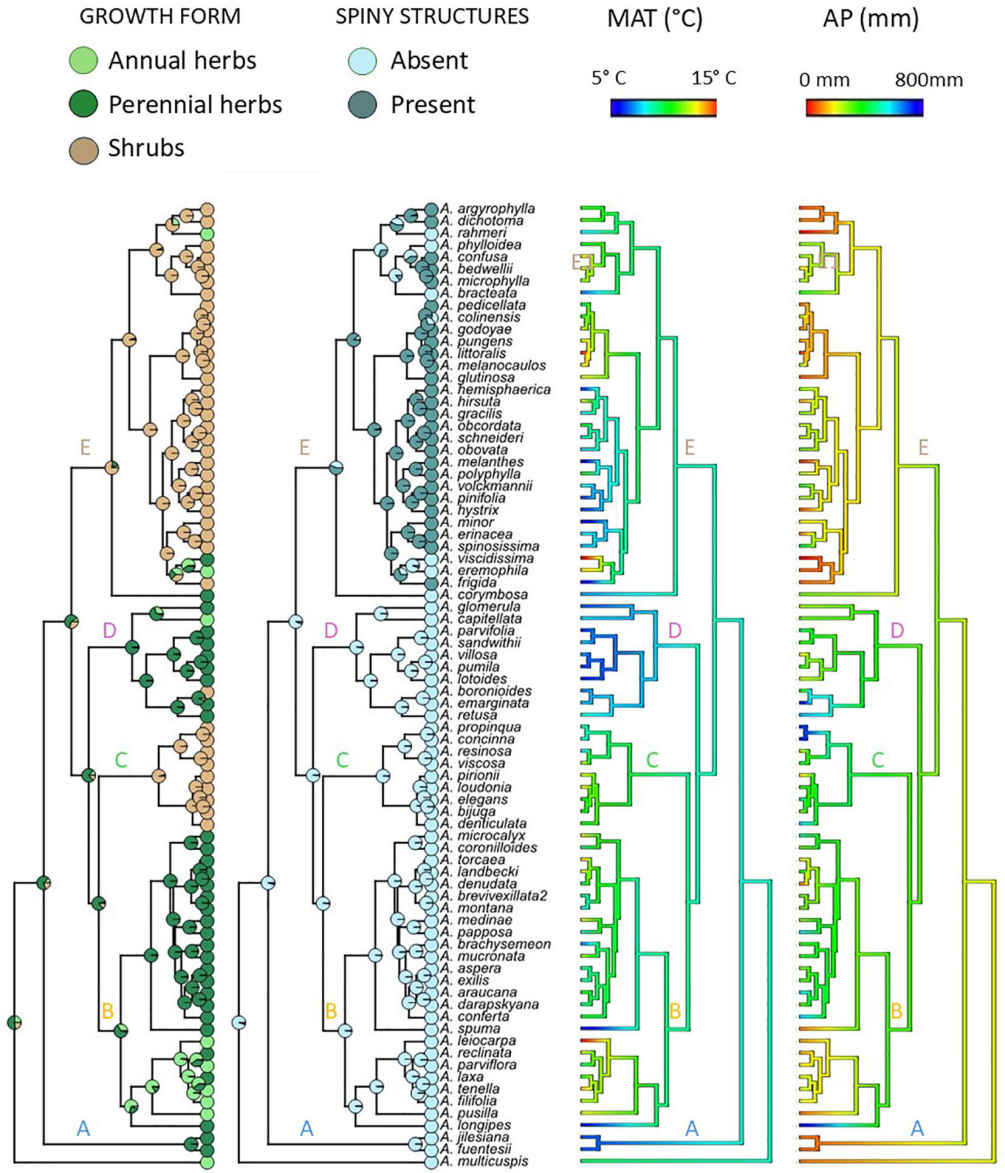
Reconstruction of ancestral states of growth forms, presence/absence of spiny structures and climatic niche. Ancestral reconstruction of growth form and presence/absence of spiny structures were performed using the Markov Chain Monte Carlo (MCMC) approach implemented in BayesTraits v4. Pie diagrams at nodes show the average probabilities of the possible states of growth form (annual, perennial herb and shrub) estimated from the 1,000 sampled traits to take into account phylogenetic uncertainty. Ancestral reconstruction of weighted mean of mean annual temperature (MAT) and mean annual precipitation (MAP) obtained from were performed PNO using a maximum likelihood method for continuous traits under a lambda model.

Similar topologies were obtained by BEAST (BE) and MrBayes (BI) analyses. In both approaches *A. multicuspis*, an annual herb that grows in northern Chile, was positioned as the sister to the rest of the genus. Five clades (named Clades A to E; see **Figure 2**) were revealed in both analyses. Clade A includes the perennial herbs *A. fuentesi* and *A. jilesiana*, which occur in northern and central Chile at high elevations (up to 4000 m). Clade B (PP: BI=1.0; BE=1.0) is formed by annual and perennial herbs, which are grouped into two subclades: B1 (BI=0.87; BE=0.92) and B2 (BI=1.0; BE=1.0). Except for *A. longipes*, all species of subclade B1 grow in warm and arid habitats at low and middle elevations of northern and central Chile, whereas species of subclade B2 primarily grow in middle and high elevations of central Chile under colder and wetter conditions (**Figure 3**). Clade B is positioned sister to Clade C (BI=0.96; BE=1.0), which is formed by unarmed shrubs restricted to low and middle elevations of central Chile. Group D (BI=1.0; BE=1.0is formed mostly by herbs that grow in cold steppes at high elevations or latitudes. Clade E (Support: BI=0.92; BE=1.0) includes the perennial herb *A. corymbosa*, which is positioned as sister to three lineages (E1-E3). Subclade E1 (BI=0.98; BE=1.0) is formed by spiny shrubs that grow mainly in cold habitats at high elevations or latitudes, except for two herbaceous species that grow in the coastal Atacama Desert (*A. viscidissima* and *A. eremophila*). Subclades E2 (BI=1.0; BE=1.0) and E3 (BI=1.0; BE=1.0) are formed principally by unarmed and spiny shrubs that occur in warm and arid habitats at low and middle elevations of northern and central Chile. Similar topologies were obtained when ribosomal and single-copy nuclear markers were analyzed separately (see Supporting Information), although some differences emerged that could result from their varying levels of resolution. Clades A to D were well supported by the ribosomal data (BI > 0.94), while the single-copy nuclear data provided weaker support. The combined dataset offered greater support and resolution for the main clades, except for clade C, which was not supported by the single-copy data. Additionally, the combined dataset provided greater support for clades E and its subclades E1, E2, and E3 than the separate datasets.

### Evolution of growth form and climatic niche

Ancestral state reconstruction of growth form based on the 1,000 sampled trees suggests that the ancestor of *Adesmia* was a perennial herb (**Figure 3**). In 82% of them, the most likely growth form for the ancestor was a perennial herb. On average, there is a 53% chance that the ancestor of *Adesmia* was a perennial herb, a 26% chance that it was an annual herb, and a 21% chance that it was a shrub.

The annual habit originated at least five times independently, with one reversion. Only three of the five transitions to annuality matched with shifts to warmer and drier habitats (**Figure 3**), corresponding to species of subclade B1, *A. eremophila* of clade E1, and *A. multicuspis* (basal species). The other two transitions occurred at high elevations, involving the origin of *A. rahmeri* and *A. capitellata*. Shrub habit evolved independently three times: in *A. boronioides*, Clade C, and Clade E (**Figure 3**). One reversion from shrub habit to herbaceous habit was detected, which matched the colonization of Coastal Atacama Desert by *A. viscidissima* and *A. eremophila*. Ancestral reconstruction for spine presence/absence indicated that spiny structures were acquired by the ancestor of Clade E. These structures would have been lost at least once in each subclade (E1, E2, and E3) and reacquired two times in subclade E1. These results indicate that the subgenus *Acanthadesmia* is not monophyletic. Phylogenetic logistic regression analyses revealed a non-statistically significant association between life span and MAT (estimate= 0.013, p=0.23) or AP (estimate<0.001, p=0.39). Likewise, no association between woodiness and MAT (estimate = -0.009, p = 0.34) nor AP (estimate <0.001, p = 0.43) was detected. Plant height did not correlate with MAT (estimate=0.006, p=0.85) or AP (estimate=-0.004, p=0.56). Phylogenetic signal lambda values for longevity (Λ= 0.76), woodiness (Λ=1.0), plant height (Λ=0.80), wMAT (Λ=0.68) and wAP (Λ=0.65) were significantly higher than zero, indicating a moderate to strong phylogenetic signal.

### Ancestral reconstruction of area

**Figure 4:**
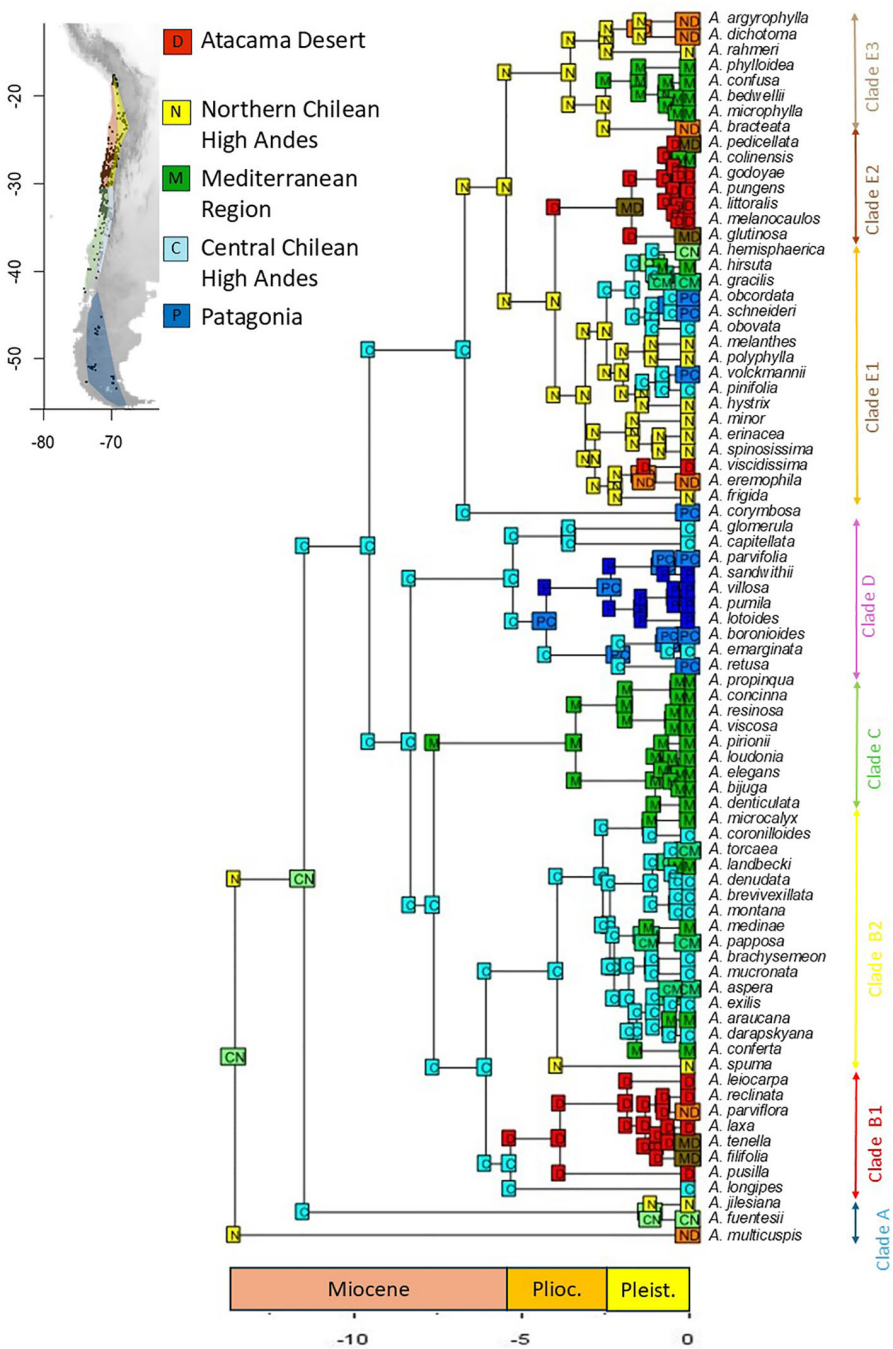
Reconstruction of ancestral distributions of *Adesmia* using the best model DEC+J (a maximum of three areas was allowed). Five areas were considered: (D) Desert, corresponding to low to mid elevations of northern Chile (18 to 30°S); (N) Northern Chilean High Andes (up to 2000 m, 18 to 30°S), (M) Mediterranean region, corresponding to low to mid elevations of central and southern Chile (30 to 40°S); (C) Central Chilean High Andes, corresponding to high elevations of central Chile (30 to 40°S); (P) Patagonia, cold steppes at high-latitudes in Patagonia (southern 40°S).

BEAST analyses indicated that the early cladogenetic events of *Adesmia* occurred approximately 10 Myr. in the Late Miocene. Ancestral range estimations under the best fit model (DEC + J; **Table 3**) suggested that the most probable ancestral area for extant Chilean species of *Adesmia* was the high Andes of northern and central Chile (**Figure 4**). Low and middle elevations of central Chile were colonized during the Late Miocene by the ancestor of the unarmed shrubs of Clade C, and later during the Pliocene and Pleistocene by several lineages of spiny shrubs and perennial herbs. The ancestor of Clade D colonized high latitudes of Patagonia during the Pliocene, followed by several lineages of spiny shrubs that extended their ranges during Pleistocene. The coastal Atacama Desert was colonized at least twice during the Pliocene, once by herbs of subclade B1 and again by spiny shrubs of subclade E2. Spiny shrubs of subclade E1 and herbs of subclade E3 colonized coastal Atacama Desert later during Pleistocene. The inclusion of the parameter “J” in the model DEC suggests that the colonization of new areas (Mediterranean region and Atacama Desert) involved multiple founder events from High Andes in northern and central Chile.

**Table 3:** Comparison of biogeographic models.

Model	j	AICc	AICc_wt
DEC	0	334.3	2.6e-05
DEC+J	0.031	313.1	1.00
DIVALIKE	0	362.8	1.7e-11
DIVALIKE+J	0.020	334	2.9e-05
BAYAREALIKE	0	414.1	1.22e-22
BAYAREALIKE+J	0.039	330	0.0002

DEC, dispersal-extinction cladogenetic model; DIVALIKE, likelihood dispersal-vicariance model; BAYAREALIKE: Bayesian biogeographical inference model; J, jump parameter that accounts for speciation processes mediated by founder events.

### Diversification analyses

**Figure 5:**
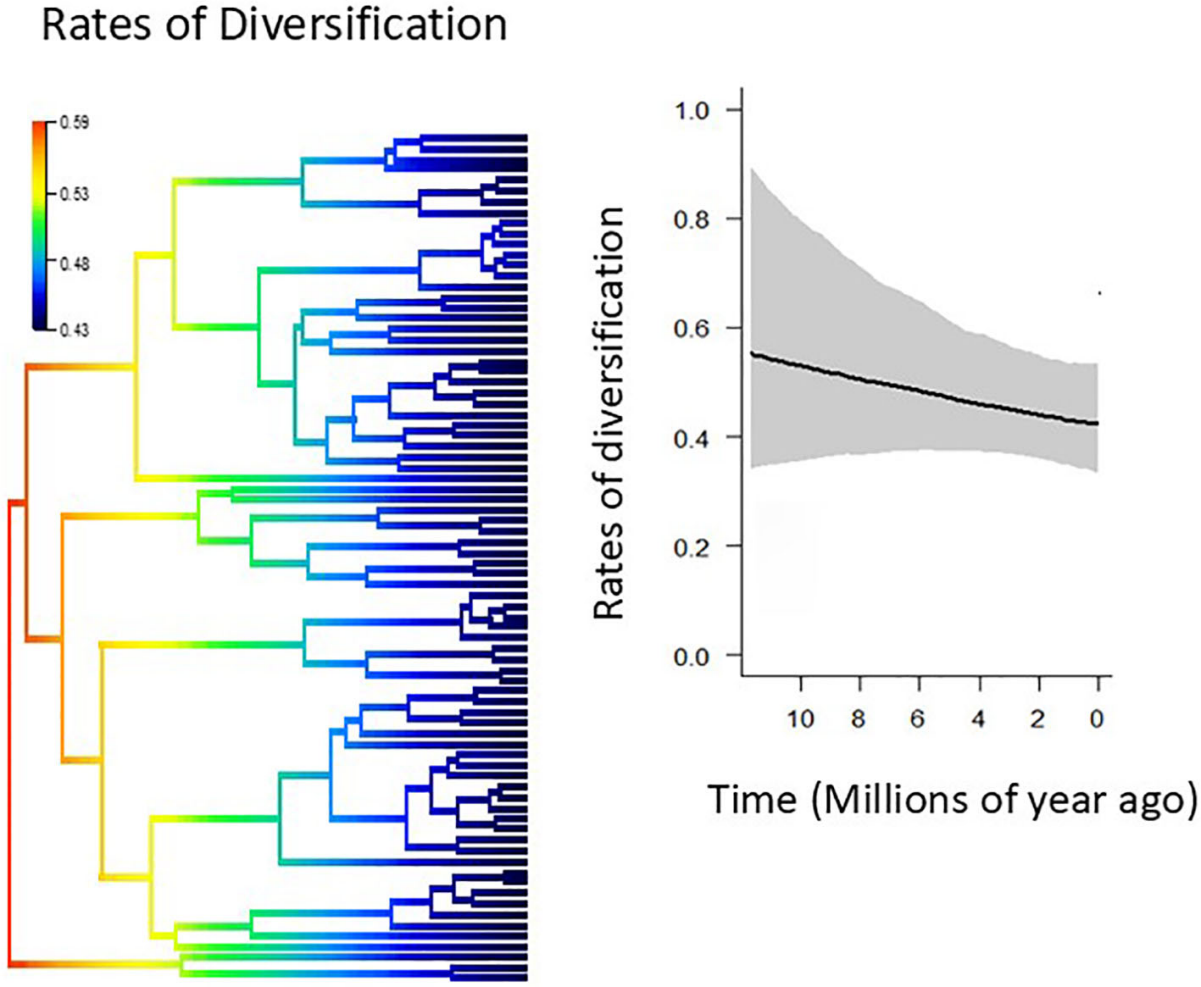
Comparison of net diversification rates through phylogeny (left) and time (right) obtained from BAMM analyses. The most probable scenario indicated that diversification rates were constant over time with no shifts (P=1.0).

The rate of diversification tends to decrease slightly over time, from a mean net diversification rate of 0.57 lineages per Myr at the root to 0.42 at the tips. However, this tendency was not statistically significant, as revealed by overlapping 95% confidence intervals (**Figure 5**). BAMM detected no significant shifts in the rate of diversification across *Adesmia*, suggesting that the high species diversity of *Adesmia* that occur in Chile is due to a uniform speciation process rather than to accelerated events of speciation.

## Discussion

Our findings suggest that the most likely growth form of the ancestor of Chilean *Adesmia* was a perennial herb, which is also the predominant growth form in its sister genera *Chaetocalyx*, *Zornia*, and *Amicia*. However, incorporating phylogenetic uncertainty makes this conclusion less certain. In 82% of the 1,000 sampled trees, the most probable ancestor of *Adesmia* was identified as a perennial herb. On average, the likelihood that the ancestor was a perennial herb (53%) was twice as high as the probability that it was an annual (26%) or a shrub (21%). Our results also suggest that the ancestor of Chilean *Adesmia* likely originated in the high Andes of northern and central Chile. The low and middle elevations of central Chile were colonized in the late Miocene. These events correlate with the last pulses of Andean uplift in northern and central Chile (Ramos and Giglione, 2008; Giambiagi et al., 2016; Garzione et al., 2017) and the intensification of aridification produced by the establishment of a phase of global climate cooling (Hinojosa and Villagrán, 1997; Hartley et al., 2005; Zachos et al., 2001).

Patagonia was colonized by perennial herbs in the Pliocene (Clade D), and later by spiny shrubs. Shifts between the high Andes and the Patagonian steppe are common (Heibl and Renner, 2012), but diversification patterns are generally consistent with south-to-north migration (Nicolas and Plunkett, 2012; Simpson et al., 2009). We found that the climatic niche associated with temperature is highly conserved in the groups that reached Patagonia (clade D and subclade E1), supporting the hypothesis that Andes acted as a corridor facilitating the dispersion of lineages that track cold conditions (Luebert and Weigend, 2014).

Atacama Desert was colonized repeatedly since the middle Pliocene, first by lineages that acquired an annual habit, and later by several lineages of perennial herbs and spiny shrubs. Multiples plant genera colonized the coastal Atacama Desert in the last 10 Mya, including *Heliotropium* sect*. Cochranea* (Luebert and Wen, 2008), *Malesherbia* (Gengler-Nowak, 2002), *Nolana* (Tu et al., 2008), *Cristaria* (Bohnert et al., 2022) and *Argylia* (Glade-Vargas et al., 2021). Different scenarios have been proposed to explain these diversification patterns. Glade-Vargas et al. (2021) suggested that intensification of aridification during the late Miocene and Pliocene, together with the elevation of coastal range during Pleistocene, generated a biogeographic corridor along the Pacific Coast. According to this hypothesis, we observed a close relationship between coastal Atacama and Mediterranean species from subclades E2 and E3. However, we also identified a close relationship between species from different elevations, indicating that east-west migrations also occurred. Activation of east-west oriented canyons during the brief pluvial phases that interrupted hyperarid conditions in the Pliocene and Pleistocene of northern Chile (Jordan et al., 2014) might have facilitated species migration. Recently Bohnert et al. (2022) showed that *Cristaria* lineages colonized different habitats in the Atacama Desert during these pluvial phases.

*Adesmia* showed multiple and bidirectional transitions between growth forms that are non-significantly associated with climate. Interestingly, annuals, perennial herbs, and shrubs can coexist under the same climatic conditions. This pattern has also been described by Hjertaas et al. (2023), who suggest that similar environments can select for different life history strategies, or alternatively, that the evolution of life strategies can be constrained by historical and developmental factors. Cousins-Westerberg et al. (2023) also failed to detect a relationship between cold tolerance and growth form in Salicornieae. These results also parallel findings in *Aeonium* (dos Santos et al., 2022), but in this case, the authors demonstrated that growth forms modulate the response of leaf size, height, and other plant traits to climate rather than being adaptations per se. Our results contrast to other comparative studies indicating that climate strongly influences the evolution of annuality (Friedman, 2020; Boyko et al., 2023), plant height (Bliss, 1971; Korner, 2003; Zanne et al., 2014), and woodiness (Howard et al., 2019; Klimes et al., 2022).

The shrub habit evolved at least three times, with two reversions to the herbaceous form. Across angiosperms, multiple transitions between herbaceous and woody habits have occurred (Klimes et al., 2022), encompassing varying degrees of woodiness, from extensive wood production (in shrubs or trees) to reduced secondary growth limited to the basis of stems (typically seen in perennial herbs) or to fascicular areas (Rowe and Paul-Victor, 2012; Trueba et al., 2015). Woodiness has been observed in island (Dulin and Kirchoff, 2010; Zizka et al., 2022) and continental lineages (Kidner et al., 2016), where it evolved in response to multiple factors such as drought resistance (Frankiewicz et al., 2021; Klimes et al., 2022), extended plant longevity resulting from a stable climate or reduced herbivory (Zizka et al., 2022), and adaptation to toxic soils (Kidner et al., 2016). Conversely, the herbaceous habit is linked to tolerance to frost and shade (Klimes et al., 2022).

The annual habit originated five times independently from a perennial herbaceous ancestor. Three transitions matched events of desert colonization, in agreement with other comparative studies that show than annual form is favored in warm and arid environments (Evans et al., 2005; Friedman and Rubin, 2015; Pérez et al., 2020). However, it also has been suggested that annual habit might evolve under other seasonally stressful conditions, such as flooding and erosion (Hjertaas et al., 2023) or frosty seasons (Kadereit et al., 2007) as an escape strategy. Accordingly, the annual habit in *Adesmia* also evolved twice in the High Andes, where short growing seasons and repeated frosts are thought to inhibit seedling establishment (Bliss, 1971; Korner, 2003; Givnish, 2015). Warm and moist soils under the snowpack is also expected to difficult the persistence of seed banks in high elevations (Hjertaas et al., 2023). The evolution of the annual habit in *A. capitellata* is accompanied by the acquisition of autonomous selfing and reduced allocation to floral structures (Arroyo and Uslar, 1993), suggesting that the annual strategy might have evolved as part of a rapid development strategy that copes with the impoverished and variable conditions for animal pollination of high Andes (Arroyo et al., 2006).

We also detected at least one reversion from the annual form to the perennial form. Transitions from perennial to annual habitat have occurred thousands of times in flowering plants, but reversions are less frequent (Friedman, 2020; Boyko et al., 2023; Hjertaas et al., 2023). Derived perennial habits have been described, for example, in Orobanchaceae and Montiaceae (Tank and Olmstead, 2008; Ogburn and Edwards, 2015).

BAMM analyses indicated a constant rate of diversification, suggesting that the high species diversity of Chilean *Adesmia* is due to uniform speciation rather than to accelerated events of speciation. No evidence of radiations was also found in *Oxalis* (Heibl and Renner, 2012), which colonized Andean and desert habitats several times. Accelerated speciation events have been detected in lineages that acquired a perennial habit, which acted as a key innovation in high elevation habitats (Drummond et al., 2012; Hughes and Atchison, 2015; Nevado et al., 2016). In contrast, our data indicates that shifts in growth form in *Adesmia* did not lead to an increase in speciation. Furthermore, perennial herbs and spiny shrubs shifted repeatedly between low and high elevations habitats, suggesting that these are successful adaptations to survive both drought and unfavorable cold seasons. However, it is important to highlight that our study sampled only the 64% of Chilean *Adesmia* species. According to Cusimano and Renner (2010), sampling less than 80% of the species can introduce biases toward downturns. These biases are particularly pronounced when deeper nodes are oversampled. Nevertheless, we believe this issue does not significantly affect our study because we made a deliberate effort to include species representing the full range of morphological and taxonomic diversity within Chilean *Adesmia*.

It is also important to note that our study only sampled the 34% of the species in the genus. Little is known about phylogenetical relationships of the remaining species of *Adesmia*, and accordingly, expanding the sample size could potentially alter the outcomes of this study, particularly the ancestral reconstruction of area and growth form. A phylogenetic study has been conducted for Brazilian species of sect. psoraleoides (Iganci et al., 2013). This study, which included some species from other regions, found a north to south pattern of diversification, suggesting an expansion from arid regions to Patagonia, followed by colonization of southern Brazil. These results indicate that Chilean species might be paraphyletic.

Our phylogenetic analysis based on five nuclear regions of Chilean *Adesmia* does not match Burkart (1967) classification of *Adesmia* into two subgenera: *Acanthadesmia*, which groups spiny shrubs, and *Adesmia*, which groups unarmed forms. Ancestral reconstruction for spine presence/absence suggest that spiny structures were acquired by the ancestor of Clade E. These structures would have lost at least once in each subclade (E1, E2 and E3) is not monophyletic. Discrepancy between morphological and molecular data was also detected by Iganci et al. 2013. Most series described for *Adesmia* by Burkart (1967) were also not recovered in the molecular phylogeny of Chilean *Adesmia*, indicating that the systematics of the genus should be revisited.

Overall, our results showed that growth forms are evolutionarily labile in *Adesmia*. However, contrary to expectations, transitions were not significantly associated with climate, suggesting that annual, perennial herbaceous, and woody habits are alternative and successful strategies to survive unfavorable seasons in both the Atacama Desert and the high Andes. The high species diversity in these regions resulted from repeated events of colonization facilitated by adaptations that allowed plants to cope with both drought and cold.

## Data Availability

The datasets presented in this study can be found in online repositories. The names of the repository/repositories and accession number(s) can be found below: https://www.ncbi.nlm.nih.gov/genbank/, Numbers in **Table 1**.
