# Supplementary figures and images for: Speciation and evolution of growth form in Adesmia D. C. (Dalbergieae, Fabaceae): the relevance of Andean uplift and aridification
Source: Front Plant Sci. 2024 Oct 15;15:1403273. doi: 10.3389/fpls.2024.1403273 (PMC11518719; doi:10.3389/fpls.2024.1403273)

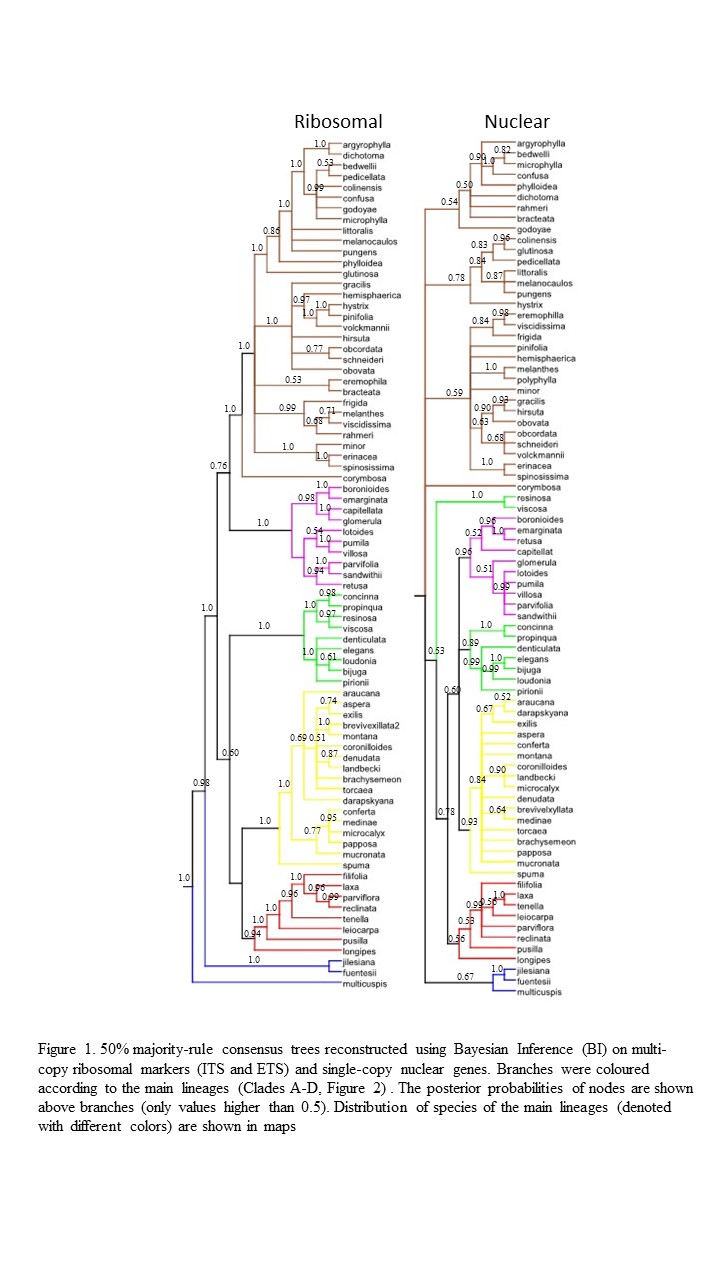

Supplement: Supplementary file 3 [file Image1.jpeg]
